# Mobile Augmented Reality as a Feature for Self-Oriented, Blended Learning in Medicine: Randomized Controlled Trial

**DOI:** 10.2196/mhealth.7943

**Published:** 2017-09-14

**Authors:** Christoph Noll, Ute von Jan, Ulrike Raap, Urs-Vito Albrecht

**Affiliations:** ^1^ PL Reichertz Institute for Medical Informatics Hannover Medical School Hannover Germany; ^2^ Universitätsklinik für Dermatologie und Allergologie Klinikum Oldenburg AöR Oldenburg Germany

**Keywords:** problem-based learning, cellular phone, education, medical, mHealth

## Abstract

**Background:**

Advantages of mobile Augmented Reality (mAR) application-based learning versus textbook-based learning were already shown in a previous study. However, it was unclear whether the augmented reality (AR) component was responsible for the success of the self-developed app or whether this was attributable to the novelty of using mobile technology for learning.

**Objective:**

The study’s aim was to test the hypothesis whether there is no difference in learning success between learners who employed the mobile AR component and those who learned without it to determine possible effects of mAR. Also, we were interested in potential emotional effects of using this technology.

**Methods:**

Forty-four medical students (male: 25, female: 19, mean age: 22.25 years, standard deviation [SD]: 3.33 years) participated in this study. Baseline emotional status was evaluated using the Profile of Mood States (POMS) questionnaire. Dermatological knowledge was ascertained using a single choice (SC) test (10 questions). The students were randomly assigned to learn 45 min with either a mobile learning method with mAR (group A) or without AR (group B). Afterwards, both groups were again asked to complete the previous questionnaires. AttrakDiff 2 questionnaires were used to evaluate the perceived usability as well as pragmatic and hedonic qualities. For capturing longer term effects, after 14 days, all participants were again asked to complete the SC questionnaire. All evaluations were anonymous, and descriptive statistics were calculated. For hypothesis testing, an unpaired signed-rank test was applied.

**Results:**

For the SC tests, there were only minor differences, with both groups gaining knowledge (average improvement group A: 3.59 [SD 1.48]; group B: 3.86 [SD 1.51]). Differences between both groups were statistically insignificant (exact Mann Whitney U, U=173.5; *P*=.10; r=.247). However, in the follow-up SC test after 14 days, group A had retained more knowledge (average decrease of the number of correct answers group A: 0.33 [SD 1.62]; group B: 1.14 [SD 1.30]). For both groups, descriptively, there were only small variations regarding emotional involvement, and learning experiences also differed little, with both groups rating the app similar for its stimulating effect.

**Conclusions:**

We were unable to show significant effects for mAR on the immediate learning success of the mobile learning setting. However, the similar level of stimulation being noted for both groups is inconsistent with the previous assumption of the success of mAR-based approach being solely attributable to the excitement of using mobile technology, independent of mAR; the mAR group showed some indications for a better long-term retention of knowledge. Further studies are needed to examine this aspect.

**Trial Registration:**

German Clinical Trials Register (DRKS): 00012980; http://www.drks.de/drks_web/navigate.do? navigationId=trial.HTML&TRIAL_ID=DRKS00012980 (Archived by WebCite at http://www.webcitation.org/ 6tCWoM2Jb).

## Introduction

According to authors such as Johnson et al [1] and Kroeker [2], augmented reality (AR) will become one of the major user interfaces of the 21st century. AR allows real and virtual objects to coexist and interact in the same space and time [3]. Using AR, virtual information can be interwoven with reality, which leads to an augmentation of the physical environment. Thanks to the ready and still growing availability of smartphones and tablets and their ever-increasing processing power, AR can now be used in a mobile manner (ie, mobile Augmented Reality [mAR]) as well. Whereas previously, AR was mainly of relevance for entertainment, marketing, or video games, it is now also entering the challenging field of teaching and training. One significant benefit of mAR for learning is the ease of modeling objects and presenting them to learners in real-world settings, so that they can get a clear idea about what they are to learn [4], and there are various studies evaluating the effects this technology has on the learning process for various user groups and settings [4-7].

In preparatory work done at Hannover Medical School, there was already an initial investigation into the possible uses of mAR for teaching and learning in a medical education setting [6]. For this purpose, a *mobile Augmented Reality blended learning environment* (mARble) app was built, which was then evaluated in comparison with conventional learning material (textbook), specifically with respect to its learning efficiency [6]. Despite the low number of cases (n=10) for that pilot study, it was possible to show positive activation for those participants who had been learning with the mAR app, and when checking the participants’ knowledge gain, the mAR group performed significantly better than those who had learned with the conventional textbook material [6].

However, it remained unclear whether this activation had to be attributed to using a different medium and its exciting novelty. Initially, it was unclear to what extent mAR had actually contributed to the learning success, a problem also mentioned by Radu [4] when contemplating the effects of different media—with entirely different means of presentation—on learning. With this study, we wanted to address this issue.

## Methods

### The Learning Environment mARble

The iPhone operating system (iOS, Apple Inc)-based app mARble-Derma (mARble-dermatology) was developed at the Peter L. Reichertz Institute for Medical Informatics of Hannover Medical School, in collaboration with Ulrike Raap, formerly of the Clinic for Dermatology and Allergy at Hannover Medical School, and her team at the clinic. It provides users with learning content organized in the form of digital flashcards. Using paper-based markers that can be placed on the skin of users, the app employs AR to recall content linked to the markers, overlay it on images of the environment if desired, and to thus add an entirely new level of information [6]. The app’s code and its content are kept separately. Via an extensible markup language-based file format, content can easily be edited or added without changing the code [6,8].

### Learning Material

The subject of dermatology was chosen for the study, as dermatology is a specialty where visual information is of high relevance when it comes to diagnosing various skin conditions, making it ideal for AR-based scenarios. The lecturer for dermatology selected altogether five relevant topics (malignant melanoma, basal cell carcinoma, psoriasis vulgaris, bullous pemphigoid, and atopic dermatitis) from the learning catalog. The learning material for the selected topics was adapted from relevant literature [9], as well as the course material normally provided to students by the department. In close collaboration with the lecturer, it was then integrated into the app. All images originated in the department and were professionally produced for teaching purposes.

### Fine-Tuning the Content: Selecting the AR Markers and Their Corresponding Content

For selecting a suitable subset out of the available markers and flashcards, a randomized single-blinded questionnaire was employed. For each of the available markers, this questionnaire contained images that had been acquired by overlaying the respective finding onto the skin of a test subject using the app. These images were then rated by 16 doctors (9 junior doctors and 7 dermatologists) working at the clinic for Dermatology and Allergy of Hannover Medical School. For each image, the doctors were asked to give a free text answer stating their diagnosis. A subsequent analysis of interrater reliability [10] led to the aforementioned selection from originally 10 markers and 6 subject areas. With one exception, only markers that were correctly recognized and had shown an interrater reliability of at least 60% were included. The marker and the subject area for “atopic dermatitis” (item 2) were included despite poor reliability (46% [6/13]); whereas location is an important aspect when diagnosing this condition, it could not adequately be deduced from the presented image. It is to be expected that with a more carefully chosen view better depicting the location, the association of the presented image with the correct diagnosis would have been more reliable, as the term “eczema,” which also covers “atopic dermatitis,” was often used to describe the depicted finding. Altogether, eight markers from five subject areas were finally included (Table 1).

**Table:** 

Item	N	Correct answers	Specialist		Incorrect answers	Specialist		Correct answers, % (n/N)
			yes	no		yes	no	
Item 1 melanoma metastases	14	9	4	5	5	2	3	64 (9/14)
Item 2 atopic dermatitis	13	6	2	4	7	4	3	46 (6/13)
Item 3 psoriasis vulgaris, single spot	16	14	6	8	2	1	1	88 (14/16)
Item 4 basal cell carcinoma, pigmented	16	3	3	0	13	4	9	19 (3/16)
Item 5 nodular melanoma	15	10	4	6	5	3	2	67 (10/15)
Item 6 basal cell carcinoma	16	16	7	9	0	0	0	100 (16/16)
Item 7 superficial spreading melanoma	14	9	2	7	5	3	2	64 (9/14)
Item 8 bullous pemphigoid	15	14	5	9	1	1	0	93 (14/15)
Item 9 urticaria	10	5	2	3	5	1	4	50 (5/10)
Item 10 psoriasis vulgaris, multiple spots	13	8	3	5	5	1	4	62 (8/13)

**Figure 1:**
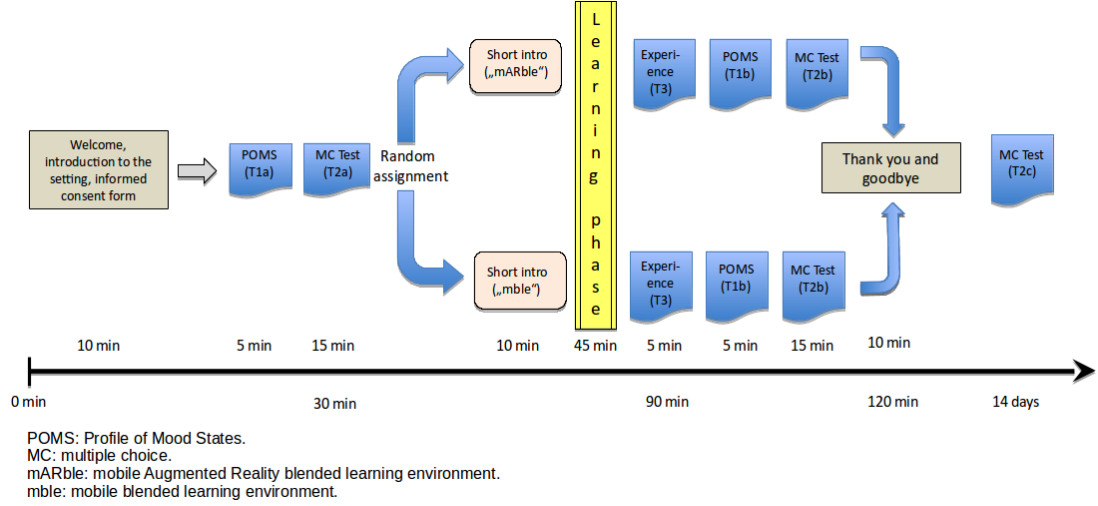
Schedule and tests that were performed. Throughout the text, individual steps or tests are referenced with the labels shown in this figure (T1a/b, T2a/b/c, and T3).

### Objective

The hypothesis to be tested in the study was that there is no significant difference in the score of correct answers (learning success) between learners who have access to mAR and those who do not. In addition, it was of interest whether there were indicators hinting at better long-term retention of acquired knowledge for those who had learned with mAR. We were also interested in whether the emotional involvement seen in the prestudy could be reproduced.

### Study

The study was conducted with approval by the institutional review board of Hannover Medical School, study number 1823-2013, amended 2014. For this study, it was decided to use the design of a two-arm, prospective randomized trial. There were two study groups, both of which were equipped with smart devices (iOS-based smartphones and tablets, specifically iPads, iPad Mini tablets, iPhones 4, or iPhones 5) with preinstalled copies of the mobile learning environment. For both groups, the software was identical, with the exception of the mAR functionality, which was only provided to one group (Figure 1).

#### Sample Size Calculation

Experiences from our previous study [6] had shown that recruiting students for extracurricular activities such as participation in a study is extremely difficult. We therefore decided to take a conservative approach in our calculations, leading to a reasonable (and realistically obtainable) number of participants while still keeping the power at an acceptable level. On the basis of our previous results [6], the sample size required for Mann Whitney *U* testing (unpaired rank sum, two-sided, effect size *d*=0.73, Laplace distribution, minimum power of .8) was calculated with G*Power 3.1 (Heinrich-Heine-Universität Düsseldorf) [11,12], leading to 21 individuals per group (altogether 42 participants). However, we chose to recruit 2 additional candidates to be able to compensate for spontaneous dropouts, at least for the initial part of the study.

**Table 2:** Descriptive statistics of the participants (N=44).

Group	Gender	n	Age in years, mean (SD^a^)	iPhone owners	Other smartphone or tablet
A	Female	9	21.45 (2.39)	9	14
	Male	13			
B	Female	16	23.05 (3.97)	16	8
	Male	6			

^a^SD: standard deviation.

#### Study Population

A total of 44 third-year medical students (25 females, 19 males, mean age=22.25 years [SD 3.33]) were included in the study. None of them had previously finished the dermatology module (Table 2).

#### Implementation

After all the participants had given their consent to being included in the study, they were given a brief introduction into the study’s topic and its schedule. Following this initial introduction, the participants were allocated to the two study groups by letting them choose a random envelope containing information about their assignment to one of the two groups, their individual study ID, and the questionnaires used in the study. These envelopes had been prepared by the study team beforehand. These were sealed, with no labeling or other discernible markings on the outside that could have provided a hint as to their specific content, and they were also mixed before being presented to the participants. Before the students opened their chosen envelopes, it was not possible for either the students or the study team to determine which group assignment was given by the envelopes’ contents.

To assess the initial emotional status of the students, the participants were asked to fill out a German version of the “Profile of Mood States” (POMS) questionnaire [13,14]. As shown in Figure 1, they were given 5 min for answering this questionnaire (T1a). To obtain a baseline about their knowledge regarding the subject areas, they were also asked to answer a single choice (SC) test consisting of 10 questions (T2a), for which they were given 15 min.

**Figure 2:**
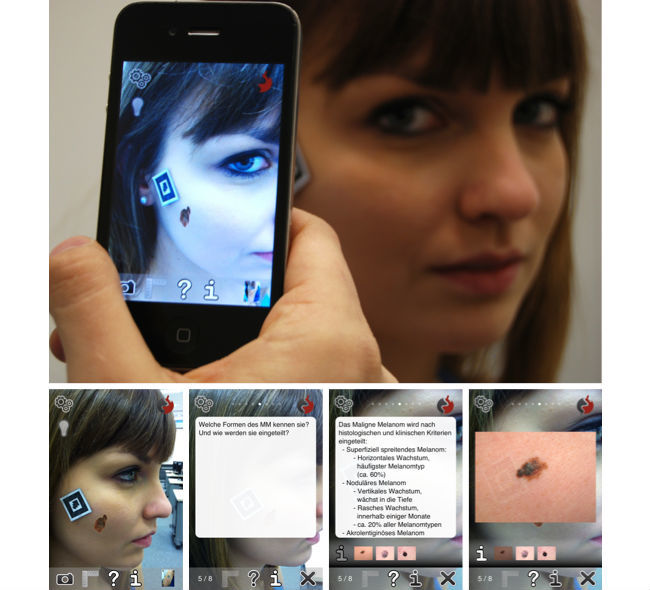
Upper row: Using mobile Augmented Reality (mAR), a malignant melanoma is simulated on the cheek of a student. Lower row: Screenshots taken within the mobile Augmented Reality blended learning environment (mARble) app. Left to right: Overlaid image, question side of a flashcard, answer side with links to additional image material, and a presentation of said image material. For the control group B, the app was provided without the mAR component, and they were solely able to access the flashcard-based information.

Whereas the setting was otherwise identical, group A learned with mAR and group B without the mAR component. Both groups were led into two different rooms where they were again given a brief introduction, this time into the basic operation of the app mARble (Figure 2). The students were then equipped with mobile devices (one per individual) on which the app had been preinstalled. They also received headphones for individual use. The participants were then allowed to study using the app for a time span of 45 min (Figure 1). Group B simply used the flashcard-based material containing textual information as well as corresponding images (Figure 2). Members of group A were given the opportunity to use the additional markers (Figure 2), for example, to place them on their own bodies, to view the corresponding findings overlaid on their skin, and to quickly access the same textual as well as image data as group B. All participants learned at their own pace. For both groups, members of the study team were present to quietly observe the learning process and to be able to react to potential technical problems.

The control group B was provided with the (content-wise) same app as group A, but the members of this group were not given any markers that they could have used to trigger the mAR-based functionalities of the app. They were only told about how they could access the provided content (flashcards) using the app’s navigation menu (Figure 2). During the learning phase, the participants of group B were allowed to learn at their own pace, without any interaction with other members of their group, and to take notes on paper if they wished to do so. Following the learning phase, the participants were asked to complete a questionnaire (AttrakDiff 2, T3) covering user experience-related aspects of what they had just experienced [15]. They were also asked to once again fill out the POMS questionnaire (T1b) about their emotional status. For filling out both questionnaires, they were given 10 min. Finally, to determine how much they had learned, they were once again asked to answer the SC test consisting of 10 questions, with the questions being presented in a random order (T2b).

Similar to group B, group A was briefed about using the app, the included flashcards, as well as attachments. Additionally, they were familiarized with using the paper-based markers serving as triggers for the mAR-based functions of the app. The participants were asked to use all of the provided eight markers for the five subject areas by placing them on their own skin and to also utilize the markers for “help” and “contact.” For two subject areas, multiple markers were available. There were three markers for “malignant melanoma” and two markers for “psoriasis vulgaris.” Similar to group B, for the study phase of 45 min duration, group A was asked to study individually, without any interaction with other members of their group, and taking paper-based notes was also allowed. After finishing the learning phase, again, similar to group B, they were also asked to complete the user experience (AttrakDiff 2, T3) and POMS questionnaires (T1b), as well as the 10-question SC test (T2b) about the five subject areas they had learned.

After reminding the participants about the Web-based follow-up survey (T2c), planned for 14 days after the day the study had taken place, everyone was thanked and the study group B were informed that they would get an opportunity to experience the full functionality of mARble, including mAR, at a later date if they so desired.

For the follow-up survey (T2c), the participants were invited via an email that contained an individual link, leading them to a Web-based version with the same questionnaire as before, consisting of 10 questions presented in a random order.

### Evaluation Tools

#### Emotional Involvement (T1a+T1b): POMS Questionnaire, German Version

Similar to the previous study by Albrecht et al [6,8], before and immediately after the learning phase, the emotional status of the students was evaluated based on the POMS questionnaire [13]. It was applied in its German, slightly modified version, as described by Biel et al [14]. This questionnaire contains 35 adjectives that can be divided into groups associated with four different emotional states, that is, fatigue–inertia (14 items), vigor–activity (7 items), tension–anxiety (7 items), and depression–dejection (7 items). Ratings are assigned based on a 7-point rating scale representing the experienced intensity (ranging from “not at all” to “very strongly”).

#### Learning Success: Single Choice Tests (T2a, T2b, and T2c)

The learning outcome was evaluated by means of the aforementioned paper-based SC tests (single choice answers) consisting of 10 questions. There were 88 test forms, with questions and answers being presented in a random order. For the follow-up survey, a Web-based questionnaire was used, which participants were able to access using their participant ID as well as a password they had received at the beginning of the study. As the participant IDs had been randomly assigned to the students—the IDs and corresponding passwords were noted on a slip of paper in the envelope the students had chosen themselves at the beginning—it was not possible to identify individual students.

The questions employed for testing closely followed the methodology also used in official exams for medical students as they are compiled by the German Institute for Medical and Pharmaceutical Examination Questions (Institut für medizinische und pharmazeutische Prüfungsfragen). The questions’ language and content were adapted to reflect the material provided in the lecture notes available for the dermatology and allergy class at Hannover Medical School, and they were checked for correctness and solvability by the module’s lecturer. The content provided by the app was also checked with respect to whether it was adequate for solving the test questions and whether it was presented in a manner that made it possible to go through all of this content within the given time frame of 45 min.

On the basis of the tests conducted before and after the learning phase, the learning efficiency (T2a, T2b, and T2c) for both groups was evaluated descriptively using the calculated mean values and corresponding SD. For hypothesis testing, a nonparametrical signed-rank test for unpaired samples was conducted (exact Mann Whitney *U* test) with Statistical Package for the Social Sciences (SPSS) version 24 (IBM Corp). All questionnaires were included in this evaluation, and each of them had been fully completed. For the follow-up survey, only those questionnaires were included in the evaluation that had been completed in the time span between the start of the follow-up period (after 14 days) up to 8 days later. Missing questionnaires were replaced by the mean values calculated for the respective group.

#### Learning Experience (T3): AttrakDiff 2

Isleifsdóttir et al [16] describe “user experience” as an important factor to consider when designing software. In their previous, preliminary evaluation of mARble, Albrecht et al [6] employed the AttrakDiff 2 questionnaire as described by Hassenzahl et al [15,17,18] to evaluate this aspect of the app. It uses altogether 28 questions, covering four different aspects (pragmatic quality, PQ; hedonic quality (HQ)-stimulation, HQ-S; hedonic quality-identification, HQ-I; and attractiveness, ATT), with 7 questions per group. For each item, semantic differentials are used, with opposite adjectives (eg, “good–bad” and “confusing–clear”) being placed at the poles of a 7-point Likert scale. In the work presented here, using a similar setting, the AttrakDiff 2 questionnaire was again used to evaluate the app’s ATT, as well as its hedonic and pragmatic qualities.

For each of the 28 attributes included in the questionnaire, mean values as well as corresponding SDs were calculated for the ratings given by the participants. For each dimension, average ratings were calculated and plotted for clarity. The values for PQ (on the x-axis) were plotted against those obtained for HQ (aggregated from the values obtained for hedonic stimulation and hedonic identification, placed on the y-axis). By including the corresponding confidence intervals into the plot, rectangles are shown that allow asserting to what extent the user experiences between both groups differ or overlap.

#### Analyzing User Behavior Based on Log Files Recorded on the Devices

To provide insights into how the participants had learned, the usage of markers as well as the included flashcards were tracked via the logging functionality integrated into the app. The recorded data included the date and time at which a marker or flashcard had been used, the type of the event (marker in focus, flashcard being invoked), the title of the marker or flashcard being used, as well as the duration of the event in seconds. As there were multiple flashcards per subject, for the flashcards, a numeric identifier was recorded as well. It was also noted whether the answer or question side of the flashcard had been displayed.

For all participants, the log files recorded during the learning phase were transferred to a central database. How long the provided flashcard content had been utilized (median values and interquartile range [IQR]) was then calculated for each group, in aggregated form as well as per flashcard (stratified for questions and answers) and per participant. For group A, median values and IQRs for the markers were calculated as well.

## Results

### Item Analysis: Single Choice Tests (T2a, T2b, and T2c)

The three SC tests were subjected to an item analysis to determine their difficulty and selectivity. For both groups, for each of the questions in a test, a difficulty index *p* was calculated with the following formula:

*p=N*_C_*/ N*

(*N*_C_=number of participants with a correct answer, *N*=number of participants in the group). A selectivity index *r* as point-biserial correlation (r_p.bis) was calculated for each test as well.

For the pretest T2a, *p* was .7682 for group A and .7782 for group B. Thus, initially, the overall difficulty for both groups was almost identical, despite differences on a per-question level, which, however, is to be expected to be able to discriminate between high and low performing participants [19]. Overall, over the course of the study, *p* rose for both groups, denoting decreasing difficulty. Directly after the initial learning phase, *p* was .8400 for group A and .8555 for group B. At the time of the final follow-up test, there were again only minor negligible differences between both groups with *p*=.8667 (group A) and *p*=.8650 (group B).

### Learning Success: Single Choice Test (T2a, T2b, and T2c)

Immediately after the learning phase (post 1, T2b), as well as after 2 weeks (post 2, T2c), both groups showed improvements compared with their initial level of knowledge (baseline, T2a). Although there were only minor differences between both groups immediately following the learning phase, with the average number of correctly answered questions rising by 3.59 (SD 1.48) for group A and 3.86 (SD 1.51) for group B (difference 2.7% between both groups), the differences between the two groups were statistically insignificant (exact Mann Whitney *U*, *U*=173.5, *P*=.10, *r*=.247).

**Figure 3:**
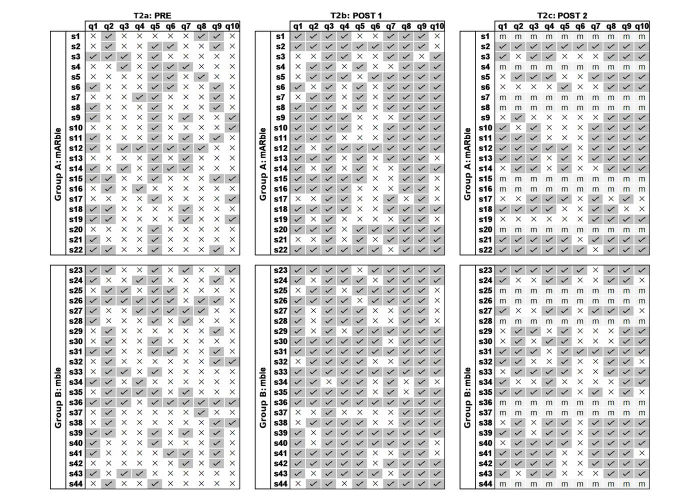
Results of the single choice tests for both groups. For the third test, missing values are denoted by m.

Descriptively, at the time of the final test after 2 weeks (Table 3 and Figure 3), both groups did not do as well as before. However, those who had learned with mAR (group A) made an average of 8.1% fewer errors compared with those who had learned without the benefits of mAR (group B).

**Table 3:** Results (number of correctly answered questions) and changes for the single choice tests administered during the study.

Phase	Group A (mARble^a^)		Group B (mble^b^)	
	Mean (SD^c^)	Change to the previous phase	Mean (SD)	Change to the previous phase
Pre (T2a)	3.41 (1.33)	-	3.91 (1.90)	-
Post 1 (T2b)	7.00 (1.48)	+3.59	7.77 (1.51)	+ 3.86
Post 2 (T2c)	6.67 (1.62)	−0.33	6.63 (1.30)	−1.14
Total (T2a to T2c)	-	+3.26	-	+2.72

^a^mARble: mobile Augmented Reality blended learning environment.

^b^mble: mobile blended learning environment.

^c^SD: standard deviation.

### Evaluating App Usage Based on Log Files Recorded on the Devices

**Figure 4:**
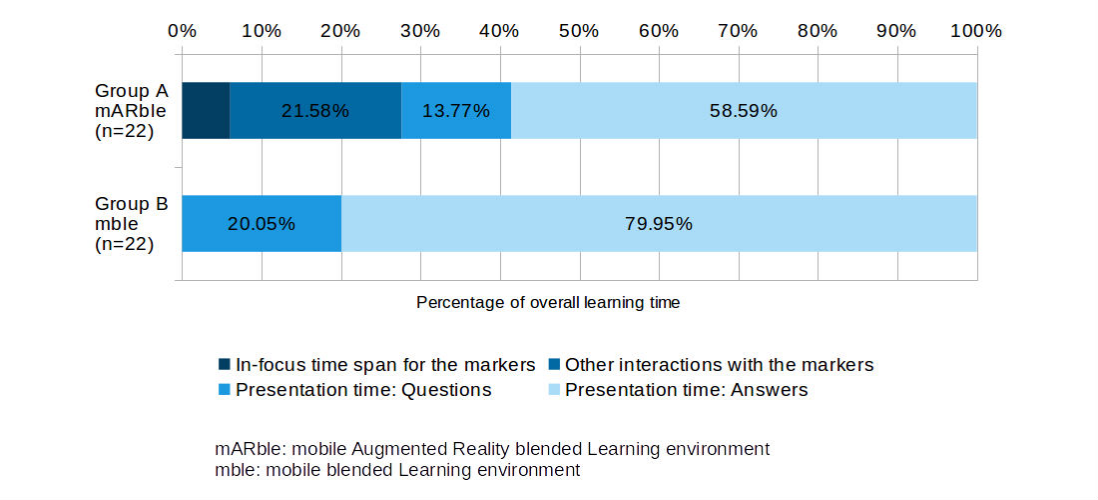
Graphical representation of the distribution of time spent on interacting with the markers as well as the flashcards, stratified for group A (mobile Augmented Reality blended learning environment [mARble]) and group B (mobile blended learning environment [mble]).

For both groups, utilization periods for the question as well as answer cards differed (Tables 4 and 5). With a total time of 42,977 s of using the flashcards (usage times for questions and answers summarized), group A used considerably less time than group B (59.816 s, see Table 4). For group A, the median usage time per flashcard was 45 s for questions and 370 s for answer cards. Altogether, each member of group A had used question cards for a median of 311 s (IQR 236 s) and answer cards for a median of 1587.5 s (IQR 503 s). For group B, the median use amounted to 71 s for each question and 245 s per answer card. Again, looking at median values, each member of group B had used the question cards for 534 s (IQR 265 s), and answers were viewed for 2094 s (IQR 874 s), both time spans again being longer than those of group A (Table 5). The lower utilization times recorded for group A can be explained by the additional effort required by interacting with the markers (in-focus time for the markers, sum for all participants: 3603 s, median time per participant 156 s, IQR 85 s; see Tables 4 and 5). Also, considerable time was spent on selecting the desired markers, placing them on the skin, focusing on them with the device’s camera etc (12,820 s, see Table 4 and Figure 4).

**Table 4:** Combined utilization times in seconds, including in-focus time spans for the markers, interaction time span, and presentation times for both question and answer parts of the flashcards, stratified by group (group A with mobile Augmented Reality [mAR], group B without mAR functionality, both n=22).

Utilization of specific parts of the application	Group A time, s (%)	Group B time, s (%)
In-focus time span for the markers, n (%)	3603 (6.07)	-
Other interactions with the markers, n (%)	12,820^a^ (21.58)	-
Presentation time: Questions, n (%)	8177 (13.77)	11,992 (20.05)
Presentation time: Answers, n (%)	34,800 (58.59)	47,824 (79.95)
Total learning time, N	59,400 (100.00)	59,816 (100.00)

^a^This value was calculated rather than measured.

**Table 5:** Utilization times in seconds (median values and interquartile ranges) for the markers (in-focus time span), marker interaction, and flashcards (questions and answers) stratified by group (group A with mobile Augmented Reality [mAR], group B without mAR functionality, both n=22).

Utilization of specific parts of the application	Group A time (s)		Group B time (s)	
	Median	IQR^a^	Median	IQR
Markers: In-focus time per participant	156.0	85.0	-	-
Presentation time per question/participant	45.0	71.0	71.0	84.5
Total presentation time per participant (questions only)	311.0	236.0	534.0	265.0
Presentation time per answer/participant	245.0	276.0	370.0	311.0
Total presentation time per participant (answers only)	1587.5	503.0	2094.0	874.0

^a^IQR: interquartile range.

### Emotional Involvement (T1a+T1b): POMS Questionnaire, German Version

**Figure 5:**
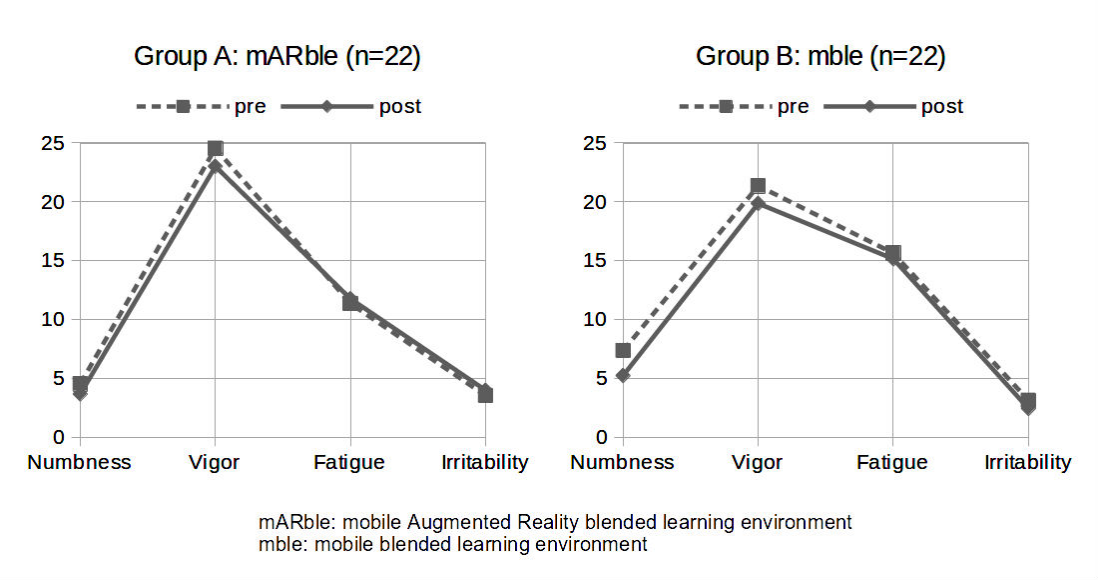
Aggregated values for irritability, fatigue, vigor, and numbness.

For the two groups, the results of the POMS tests applied before and after the learning phase with the aim of determining whether there were any changes in the participant’s emotional status did not show significant differences with respect to the evaluated qualities (see Table 6 and Figure 5). Descriptively, differences were seen for the two dimensions of “irritability” and “numbness,” whereas for both groups, “fatigue” did not change as much. For “vigor,” the decrease was almost equal for both groups (decrease for group A: 1.54, for group B: 1.5). For group B, “numbness” decreased by 2.11, from 7.36 (SD 8.54) to 5.25 (SD 7.56). This decrease was larger than for group A, where “numbness” had only been reduced by 0.87, with an initial value of 4.55 (SD 4.78), which changed to 3.68 (SD 4.52) after the learning phase. For “irritability,” there was a slight increase for group A and a slight decrease for group B.

**Table 6:** Aggregated values for numbness, vigor, fatigue, and irritability for groups A and B (both n=22).

Group	Phase	Dimensions			
		Numbness, mean (SD^a^)	Vigor, mean (SD)	Fatigue, mean (SD)	Irritability, mean (SD)
A	Pre	4.55 (4.78)	24.55 (5.47)	11.36 (5.11)	3.55 (4.70)
	Post	3.68 (4.52)	23.01 (5.02)	11.77 (6.79)	4.00 (4.35)
B	Pre	7.36 (8.54)	21.36 (6.69)	15.64 (8.64)	3.14 (3.51)
	Post	5.25 (7.56)	19.86 (6.39)	15.15 (9.47)	2.45 (3.19)

^a^SD: standard deviation.

**Figure 6:**
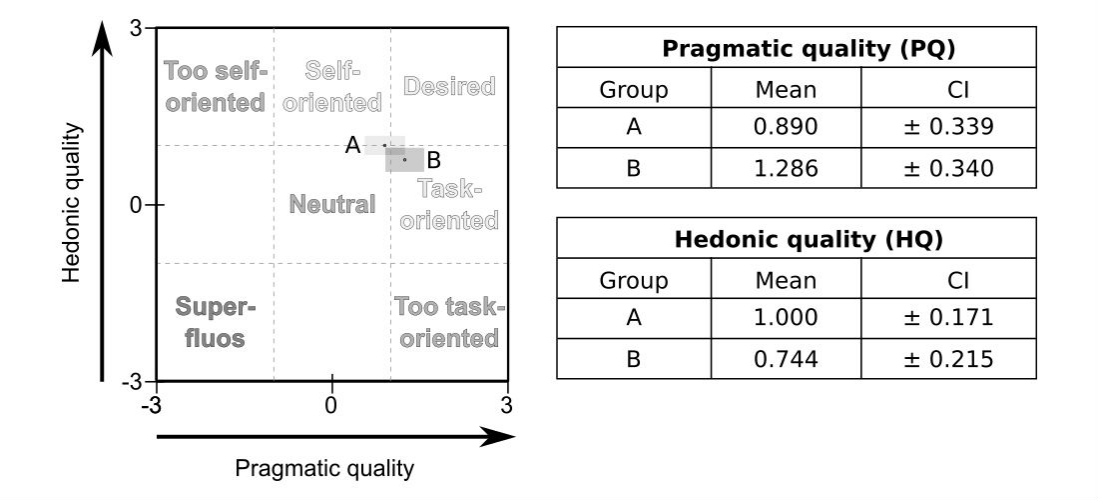
Left: Portfolio with average values of the dimensions pragmatic quality (PQ) and hedonic quality (HQ) and the respective confidence rectangles of A (mobile Augmented Reality blended learning environment [mARble]) and B (mobile blended learning environment [mble]), modified following Hassenzahl et al. Right: Corresponding values.

**Figure 7:**
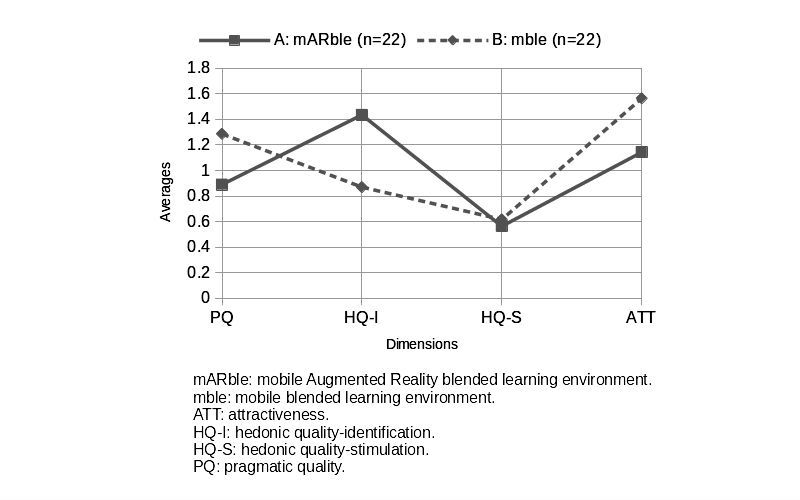
Average values calculated for the four qualities (both groups).

### Learning Experience (T3): AttrakDiff 2

The learning experience was rated positively by all participants, independent of whether they had learned with or without the mAR component, with only marginal differences (descriptive) between both groups (Figures 6 and 7,Table 7). Nevertheless, as the confidence rectangles for both groups overlap (Figure 6; [15]), this is statistically insignificant [18]. However, AR-based learning was rated better with respect to HQ, and there was also an emphasis on “self-orientation,” which can be attributed to the greater degree of self-centeredness (HQ-I) calculated for this group. In contrast, for group B, ratings emphasized the PQ of the learning experience, mirroring its perceived task-orientation. Differences between the average values calculated for PQ and HQ (aggregated from HQ-I and HQ-S) are negligible. Both groups gave similar ratings for stimulation (HQ-S), with the app without mAR being rated slightly more attractive (group A: 1.143, group B: 1.564).

**Table 7:** Aggregated values calculated for the four qualities covered by AttrakDiff 2: pragmatic quality (PQ), identification (HQ-I), stimulation (HQ-S), and attractiveness (ATT) for groups A and B (both n=22).

Group	PQ^a^ mean (SD^b^)	HQ-I^c^ mean (SD)	HQ-S^d^ mean (SD)	ATT^e^ mean (SD)
A	0.890 (0.76)	1.435 (0.51)	0.564 (0.57)	1.143 (0.60)
B	1.286 (0.77)	0.870 (0.78)	0.617 (0.66)	1.564 (0.65)

^a^PQ: pragmatic quality.

^b^SD: standard deviation.

^c^HQ-I: hedonic quality-identification.

^d^HQ-S: hedonic quality-stimulation.

^e^ATT: attractiveness.

## Discussion

### Ascertaining the Effects of mAR

The basic suitability of the mAR-based concept for teaching purposes had already been evaluated in a previous study, where a comparison between conventional learning (using textbooks) and app-based learning was presented, which also included mAR [6]. At that time, a clear advantage of the app-based approach versus textbook-based learning was shown. However, it was unclear whether the positive effects that had been noted could in fact be attributed to the AR component. There was also a suspicion that the learning medium itself, that is, the excitement of using a mobile phone or tablet personal computer (PC), might already have influenced the results [6]. On the contrary, in this study, with the learning scenarios and presentation of the learning content being identical (multimedia-supported flashcards presented on mobile phones and tablet PCs) with the exception of the mAR component, it was possible to examine the influence of the mAR component on both learning success and learning experience.

### Principal Findings

Surprisingly, the test scores showed an almost identical increase in the average number of correct answers for both groups (pre to post 1, average improvement for group A: 3.59 [SD 1.48], group B: 3.86 [SD 1.51]; exact Mann Whitney *U*, *U*=173.5; *P*=.10; *r*=.247). Therefore, simply attributing the learning success to the mAR component seems implausible. In comparison with our previous study, whether the greater increase in knowledge is simply because of the use of mobile technologies in general rather than the influence of the mAR component (with its seemingly small contribution shown here), warrants further scrutiny and needs to be considered in future work. However, indications—albeit small—of possible long-term effects may be of interest; at the time of the follow-up test 14 days later, the average number of correct answers only decreased by 0.33 (SD 1.62) for group A but by 1.14 (SD 1.30) for group B, which had not had access to the mAR component of the app while learning. Unfortunately, the dropout rate at T2c (Figure 1) was too high to permit a more confident assertion, but it may be reasonably assumed that the mAR component contributes to committing what is learned to long-term memory, and this is indeed an interesting subject to be examined in later studies. On a side note, we do not believe that repeat testing—that is, using the same tests for T2a, T2b, and T2c (Figure 1)—had a significant influence on the results. During the course of the study, none of the students were provided with either their test scores or the correct answers to the presented questions, which would have given them the opportunity to improve their results. They were only able to base their answers on the provided study material, and if any of the participants had cheated or memorized the answers based on the previously administered tests, we would have expected a more significant increase of their knowledge.

Particularly noteworthy was that group A, learning with mAR support, spent obviously much less time on using the flashcard-based content (identical for both groups) than their counterparts in group B (group A: 1587.5 s [IQR 503 s], group B: 2094 s [IQR 874 s]). Group A spent a significant amount of the allocated time on interacting with the markers, which amounted to a total of 3603 s for all participants (median marker usage per participant: 156.0s, IQR 85.0, also see Table 5). Whereas for the missing 12,820 s, there was no hard evidence proving additional marker usage in the log files (see Table 4), there were observations by the principal investigator who was present during the learning phase that there had indeed been significant mAR related interaction which—for technical reasons—had not been recorded by the app. This included time spent on searching for the desired markers, placing the markers on the skin, trying to focus on the markers, etc, which can certainly be rated as marker-related use of the app. It is up for speculation whether there is an effect of AR and interaction on the learning success that might have effects on better committing knowledge to long-term memory. Future study designs need to consider this aspect carefully. However, some indications for a potentially positive impact of interactive components on the learning process and commitment of knowledge to long-term memory can be found in literature.

In comparison to other technology-supported learning techniques, there are several mentions of potentially positive as well as negative effects of AR on the learning process [4]. In the past, there were fears that with AR demanding a higher level of focus from learners than, for example, simple multimedia supported learning modules—and possibly requiring more attention for technical aspects—AR might in fact distract students from the presented content [20]. However, we do not believe this to be true, as nowadays, when implemented in a mobile manner, on devices users are familiar with, many of the complexities previously attributed to AR are much less of an issue. This was also corroborated by observations we made during the study, where none of the participants of the mARble group indicated problems with handling the application. In fact, there were early mentions of AR and its playful aspects possibly decreasing cognitive load [21], encouraging students to be creative, to explore the provided content, and to make exciting discoveries on their own, thereby also improving learner's motivation.

The directed attention required when using AR is often also described as beneficial. AR's ability to direct its users’ attention to the relevant content, effectively highlighting important content [4,22], as well as the ability to physically enact a learning experience or at least interact with the content, may lead to enhanced memory encoding and better retention of what is being learned [4]. There are also indications that this physical interaction may activate kinesthetic schemas [23], which may also have a positive influence on the learning outcome and help with transferring acquired knowledge from working memory (with relatively low-capacity) to (high-capacity) long-term memory [24].

The learning experience for both groups was evaluated based on the method described by Hassenzahl et al [15,17,18]. Descriptively, mAR was rated more self-oriented, which was because of higher average values in the hedonic domain and smaller average values for pragmatic qualities in comparison with mobile blended learning environment (mble). Nevertheless, as the confidence rectangles for both groups overlap (see Figure 6), this is statistically insignificant [17]. In detail, both systems were rated as similarly stimulating (see Figure 7), which is consistent with ratings for mARble in the previously conducted study [6]. Thus, the stimulating effect is probably rather attributable to the app and the devices it runs on rather than to the mAR component. With respect to a possible self-oriented perception of the AR-based learning experience, the intense (and time-consuming) engagement with the mAR component may be an explanation. However, this hypothesis needs to be further corroborated by additional studies.

In contrast, there were no significant differences between both groups in the emotional realm, as evaluated by the POMS questionnaire. For “numbness,” “vigor,” “fatigue,” and “irritability,” there were only marginal differences in the ratings of both groups (see Table 6 and Figure 5).

### Limitations

As indicated, the study design was adapted according to the general difficulty of recruiting students. The highly streamlined and demanding curriculum medical students have to deal with does not give them much room for participating in activities that they perceive as further reducing their spare time. With this kept in mind, we were forced to make a compromise to the study design by calculating the sample size with a power of 0.8. For the future, for disciplines where visual content plays an important role in medical education, we will therefore aim at integrating our approach into the curriculum, thus also giving us access to a larger number of (potential) study participants.

There is room for debate about whether the random allocation of female and male participants to the two groups, which lead to a rather heterogeneous sample for both, had any influence on the results of the SC tests.

With respect to the markers, based on the chosen technical approach, it was impossible to record usage times other than those that were caused by the markers being in the camera’s focus, defined as the time span from recognition of a marker to a flashcard being displayed. Other efforts required for making use of the markers, leading up to them being in focus (selection of the desired markers, placing them on the skin, and trying to focus the camera) were not logged. In follow-up studies, a way for recording the time to fulfill these tasks needs to be found. Finally, assessing emotional involvement solely based on the POMS questionnaire is less than ideal, and care should be taken to identify an instrument better suited to evaluating the self-oriented character of the mAR-based approach.

### Conclusions

Using mobile technologies for learning purposes integrated into a multimedia-based concept, for example, with a flashcard-based approach similar to the one presented here, can be an effective approach that is at least equivalent to conventional ways of learning, if not better [6]. In this study, isolated indications for the actual impact of mAR on learning success could not be found. The effect described in the previous study may be attributable to the impact of other mobile design aspects rather than the mAR component. Larger-scale evaluations seem advisable for providing final evidence. However, whereas both groups of students obtained similar results regarding learning success, compared with their counterparts, the mARble group spent a significant part of their allocated learning time on AR-related interactions instead of on the flashcards providing textual information, pointing to the potential benefits of mAR on knowledge retention. The (descriptive) indications we found for mAR’s potentially positive influence on committing knowledge to long-term memory also point in this direction. Finally, the presented work also found indications pointing to the self-oriented character of mAR-based learning but unfortunately with a lack of significance. Whether—and if so, how—this contributes to the learning process also needs to be investigated in future studies.

## Abbreviations

AR: augmented reality
ATT: attractiveness
HQ: hedonic quality
HQ-I: hedonic quality-identification
HQ-S: hedonic quality-stimulation
iOS: iPhone operating system
IQR: interquartile range
mAR: mobile Augmented Reality
mARble: mobile Augmented Reality blended learning environment
mble: mobile blended learning environment
PC: personal computer
POMS: Profile of Mood States Questionnaire
PQ: pragmatic quality
SC: single choice
SD: standard deviation

## Appendix

Multimedia Appendix 1 Completed CONSORT checklist.
